# The report of anthocyanins in the betalain-pigmented genus *Hylocereus* is not well evidenced and is not a strong basis to refute the mutual exclusion paradigm

**DOI:** 10.1186/s12870-021-03080-9

**Published:** 2021-06-29

**Authors:** Boas Pucker, Hidam Bishworjit Singh, Monika Kumari, Mohammad Imtiyaj Khan, Samuel F. Brockington

**Affiliations:** 1 Department of Plant Sciences, University of Cambridge, Tennis Court Road, Cambridge, CB2 3EA UK; 2 Biochemistry and Molecular Biology Lab, Department of Biotechnology, Gauhati University, 781014 Guwahati, Assam India

## Abstract

Here we respond to the paper entitled “*Contribution of anthocyanin pathways to fruit flesh coloration in pitayas*” (Fan et al., BMC Plant Biol 20:361, 2020). In this paper Fan et al. 2020 propose that the anthocyanins can be detected in the betalain-pigmented genus *Hylocereus*, and suggest they are responsible for the colouration of the fruit flesh. We are open to the idea that, given the evolutionary maintenance of fully functional anthocyanin synthesis genes in betalain-pigmented species, anthocyanin pigmentation might co-occur with betalain pigments, as yet undetected, in some species. However, in absence of the LC-MS/MS spectra and co-elution/fragmentation of the authentic standard comparison, the findings of Fan et al. 2020 are not credible. Furthermore, our close examination of the paper, and re-analysis of datasets that have been made available, indicate numerous additional problems. Namely, the failure to detect betalains in an untargeted metabolite analysis, accumulation of reported anthocyanins that does not correlate with the colour of the fruit, absence of key anthocyanin synthesis genes from qPCR data, likely mis-identification of key anthocyanin genes, unreproducible patterns of correlated RNAseq data, lack of gene expression correlation with pigmentation accumulation, and putative transcription factors that are weak candidates for transcriptional up-regulation of the anthocyanin pathway.

**Supplementary Information:**

The online version contains supplementary material available at 10.1186/s12870-021-03080-9.

## Background

In the plant kingdom, betalains occur only in the order Caryophyllales where they substitute the otherwise ubiquitous anthocyanin pigments [1, 2]. Although betalains are found in most families in Caryophyllales, several families have anthocyanin pigmentation and do not produce betalains. Betalains and anthocyanins have never been found in the same species and are widely held to be mutually exclusive at the organismal level [3, 4]. However, both pigments have been observed in a genetically engineered tomato plant [5], on transgenic heterologous production of betalains. The molecular basis of mutual exclusion is unclear, especially as betalain-pigmented species seem to retain all the genes encoding the necessary enzymatic machinery for anthocyanin synthesis. It remains a remarkable and largely unexplained biological conundrum that has been reinforced by repeated observations for over fifty years [6–8].

With this as context, Fan et al. [9] recently reported anthocyanins within the betalain-pigmented genus *Hylocereus* (Cactaceae), also commonly called Pitaya. Fan et al. [9] analysed the fruits of three closely related species – a red-fleshed *Hylocereus polyrhizus, a* white-fleshed *Hylocereus undatus*, and an intermediate pink-fleshed hybrid (*H. polyrhizus x H. undatus*). Based on the analysis, they reported to correlate the accumulation of anthocyanins with the colour of red and pink fruit pulps, and the expression levels of anthocyanin biosynthesis genes. Fan et al. [9] suggest that their findings “*refute the paradigm of mutual exclusion of anthocyanins and betalains within the same species/tissue*”.

However, we have doubts about the findings of Fan et al. [9] and below, we outline our concerns.

## Main text

### No detection of betalains

Fan et al. [9] did not report the detection of betalains in the fruits of *Hylocereus* cultivars in their analyses. But as cited by Fan et al. [9], a range of betalain pigments have previously been detected by numerous studies in the same species [10–14]. Using the same two species as Fan et al. [9], earlier studies convincingly report betalain accumulation in red-fleshed *H. polyrhizus* and white-fleshed *H. undatus* [13, 14], and, also, correlated betalain accumulation with colour development at different stages of maturity of the red-fleshed species *H. polyrhizus* [12]. The first step in the analysis by Fan et al. [9] was an untargeted metabolite analysis that identified 443 different metabolites, including tyrosine, L-DOPA and *cyclo*-DOPA-5-*O*-glucoside which are intermediate metabolites in the betalain pathway - but no betalains. We do not understand why betalains were not detected in an untargeted metabolite analysis, when they have been previously shown to abundantly occur in these species [11–14].

### Profiling of anthocyanins does not meet widely held standards

Fan et al. [9] reported the detection of five distinct anthocyanins. However, they provided very little methodology with respect to the initial metabolic profiling, with only the following brief statement: “*Extract preparation, metabolite extraction, identification and quantification were performed following standard procedures of Suzhou BioNovoGene Metabolomics Platform, Suzhou, China*”. We find this to be insufficient evidence and would expect at least to see the LC-MS/MS spectra and co-elution/fragmentation of the pigments versus authentic reference compounds. This standard practice is particularly important to uphold in betalain-pigmented species where there is no prior expectation to detect anthocyanins. We believe it is also important to highlight the need for standards in metabolite analyses more widely, because studies cited by Fan et al., [9] as evidence for anthocyanins in *Hylocereus* have similar methodological limitations and are likely also not solid evidence for the presence of anthocyanins.

### Reported anthocyanins do not clearly correlate with flesh colouration

Fan et al. [9] reported that the accumulation of anthocyanins positively correlated with pink- and red-pigmented flesh indicating their “*probable contribution to flesh coloration*”. However, the reported anthocyanin Delphinidin 3-rutinoside, which is blue or pink coloured, accumulates to higher levels in the white-fleshed *Hylocereus undatus*. Indeed, based on their ion-intensity analyses (Fig. 3) the accumulation of Delphinidin 3-rutinoside is at a higher level in the white-fleshed *Hylocereus undatus* than the combined accumulation of the other 4 anthocyanins in the red-fleshed *Hylocereus polyrhizus.* Further, if all the detected anthocyanins were combined, the white pulp cultivar (*H. undatus*) would have the maximum anthocyanin content in its pulp. We therefore cannot understand why the flesh of *H. undatus* is white, given that the authors claim anthocyanins significantly contribute to flesh colouration.

### qPCR quantification is missing for key anthocyanin biosynthesis genes

Fan et al. [9] reported a significant increase in transcript abundance of genes associated with flavonoid synthesis correlated with pink- and red-coloured flesh. Genes they reported as showing this pattern include *C4H (Cinnamate 4-hydroxylase*, *F3H (flavonoid-3’–hydroxylase)*, *F3’5’H (flavonoid-3′,5′-hydroxylase)*, *DFR (dihydroflavonol-4-reductase)*, and *ANS (anthocyanidin synthase*). Fan et al. [9] provide a qPCR analysis of selected genes to support their RNAseq experiment, and which is largely concordant. However, both *ANS* and *DFR* are missing from the qPCR data. This omission is difficult to understand, as these are the two most important genes for their data interpretation, as they encode late stage enzymes in anthocyanin biosynthesis. Nonetheless, their RNAseq data reports very low transcript abundance of *ANS* in the white-fleshed *H. undatus*, which is difficult to reconcile with their report of high levels of anthocyanins in the same species. Especially as anthocyanin biosynthesis is considered a model system for regulation through transcriptional control thus a good correlation between the abundance of enzyme encoding transcripts and anthocyanins is common [15–17].

### Annotation and orthology assignment appear erroneous

The authors have deposited their raw RNAseq datasets but not their transcriptome assembly and it was not available on request. It is therefore not possible to assess their annotation directly. Nonetheless, we re-assembled their RNAseq datasets for their three taxa, with our own protocols [18]. We attempted to identify an equivalent set of anthocyanin and flavonoid biosynthesis candidate genes based on homology to previously described sequences involved in the flavonoid biosynthesis (see methods for details). The results of our annotation differ markedly from Fan et al. [9]. Most striking is that our phylogenetic analysis did not reveal a F3’5’H candidate, but strongly suggested that the only candidate is actually a F3’H. These candidates show all conserved amino acid residues expected of F3’Hs, while they lacked at least one conserved residue of F3’5’Hs. F3’5’H is a key enzyme in the biosynthetic pathway of some of their reported anthocyanins, including delphinidin. Equally striking is the absence of a true *DFR* sequence in our *H. polyrhizus* transcriptome assembly (Additional file 1). There are several putative *DFR*-like candidates, but *DFR* belongs to a large multi-gene family, and none of the putative *DFR* sequences is confirmed to be a *DFR* ortholog. When analyzing all *DFR* candidate sequences in a phylogenetic tree with previously described *DFR* sequences of other species, no sequence of the *H. polyrhizus* assembly falls into the Caryophyllales DFR clade. This last finding is important as DFR is a late-stage enzyme in the pathway to anthocyanin synthesis and *DFR* has previously been shown to have reduced and/or tissue specific expression in betalain-pigmented species [19].

### Reported transcript abundance for anthocyanin genes is not reproducible

**Fig. 1:**
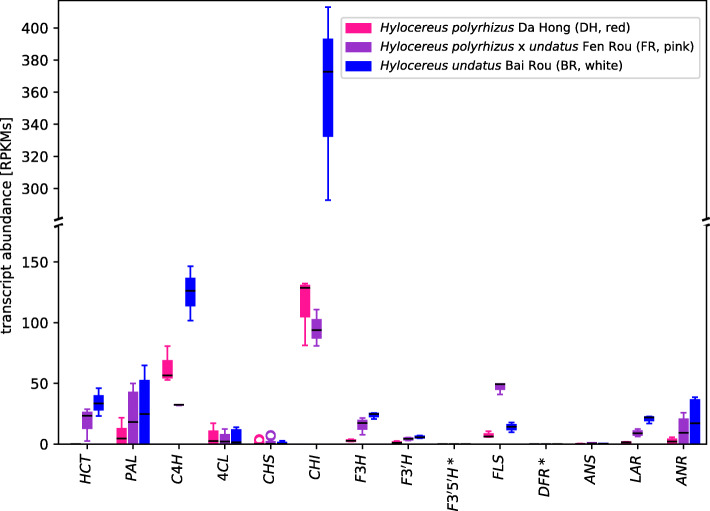
Transcript abundance on a gene set that includes all genes reported by Fan et al., [9] (and with the addition of *PAL*, *4CL* and *CHI*) presented in Fig. 5 of Fan et al. [9] and additional genes of the flavonoid biosynthesis. *F3’5’H* and *DFR* (marked with an *) were not detected in the transcriptome assembly and are therefore considered as no expression detectable. Transcript abundances of multiple isoforms or homologs were summarized per step in the pathway

We quantified transcript abundance of each homolog separately to examine their correlation across the three differently pigmented species (Fig. 1). We did not recover the same patterns of transcript abundance for anthocyanin synthesis genes as Fan et al. [9]. We find the depiction of transcript abundance in Fan et al. [9] to be slightly visually misleading, as each gene homolog is plotted individually with the Y-axis length normalised, which has the effect of under-emphasizing when genes have relatively low abundance. We therefore re-plotted all gene homologs on the same axis, to highlight that *DFR* cannot be detected in transcriptome assemblies of two of three species, and *ANS* expression in all three *Hylocereus* cultivars was negligible (RPKM < 2). In summary, from re-analysis of the transcript abundances of flavonoid and anthocyanin genes, we find no evidence to support the presence of a functional anthocyanin synthesis pathway in the fruits of *Hylocereus*, and no evidence of correlation with pigmentation in the fruit flesh (Table 1).

**Table 1:** Comparison of the *de novo* transcriptome assemblies. *BR* *Hylocereus undatus* Bai Rou, *FR* *Hylocereus polyrhizus x undatus* Fen Rou, *DH* *Hylocereus polyrhizus* Da Hong

Assembly Criteria	BR (white)	FR (pink)	DH (red)	Fan et al., [9]
Number of contigs	157,295	62,575	78,755	Not reported
Assembly size [bp]	182,080,466	52,888,161	73,229,765	49,212,589
E90N50	1841	1527	1670	Not reported
N50	1953	1330	1498	1,647
Complete BUSCOs	85.2 %	56.4 %	70.9 %	70 %

### Putative MYB regulators are not homologs of known activators of the anthocyanin pathway

**Fig. 2:**
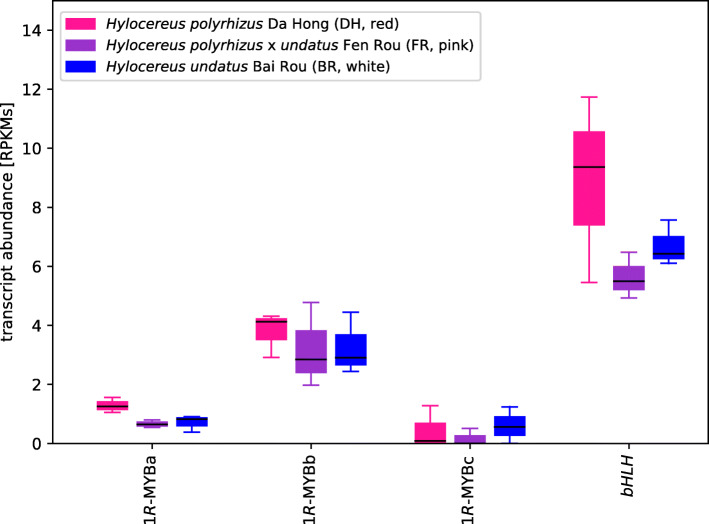
Transcript abundance of MYB and bHLH transcription factors. *1R-MYBa*, *1R-MYBb*, *1R-MYBc*, and *bHLH* were identified based on qPCR primer sequences provided by Fan et al., [9]

Fan et al. [9] discussed two MYBs and one bHLH and suggested a role for these transcription factors in the pigmentation patterns of interest. However, we found no evidence of any PAP1 R2R3-MYB homologs which typically up-regulate *ANS* [20] in our assemblies (Additional file 1). Moreover, we used the sequences of qPCR primers to recover the corresponding full-length sequences from our assemblies and found 3 corresponding MYB sequences compared to the 2 discussed by Fan et al. [9]. None of their sequences fall into the clade of R2R3-MYBs, but rather are similar to MYBS3 which have only a single MYB repeat (MYB1Rs). Single repeat MYBs have previously been reported as repressors of anthocyanin and flavonoid biosynthesis [21] rather than activation. Single repeat MYBs also do not interact with bHLH transcription factors, as do the R2R3-MYBs, so it is not clear what significance the authors are drawing from the expression of these MYBs or the bHLH gene. Finally, we quantified transcript abundance in their datasets using our assemblies and did not recover patterns commensurate with their qPCR data (Fig. 2).

## Materials and methods

### Transcriptome assembly

RNAseq datasets of different cultivars were retrieved from the Sequence Read Archive via fastq-dump [22]. Trimming and adapter removal based on a set of all available Illumina adapters were performed via Trimmomatic v0.39 [23] using SLIDINGWINDOW:4:15 LEADING:5 TRAILING:5 MINLEN:50 TOPHRED33. We decided to use separate transcriptome assemblies for the three species, because the assembly quality appears to be superior to the quality of a combined assembly. If transcripts are not recovered through this approach, it is unlikely that they have a substantial contribution to the fruit colour. Clean read pairs were subjected to Trinity v2.4.0 [24] for *de novo* transcriptome assembly using a k-mer size of 25. Short contigs below 200 bp were discarded. Previously described Python scripts [25] and BUSCO v3 [26] were applied for the calculation of assembly statistics for evaluation. Assembly quality was assessed based on continuity and completeness. Although assemblies were generated for all three species, the assembly generated on the basis of the data sets of *Hylocereus undatus* (SRR11190792-SRR11190794) was used for all down-stream analyses.

### Transcriptome annotation

Prediction of encoded peptides was performed using a previously described approach to identify and retain the longest predicted peptide per contig [25]. Functional annotation was performed by combining InterProScan5 [27] with annotation transfer from *Arabidopsis thaliana* and *Beta vulgaris* based on reciprocal best BLASTp hits [25]. Genes involved in the flavonoid biosynthesis were identified via KIPEs [28] using the peptide mode (Additional files 2 and 3). An additional tBLASTn [29] search with DFR peptide sequences was performed to screen for a putative degenerated DFR transcript which could have been missed in the BLASTp search. Predicted peptide sequences were also screened via KIPEs to identify MYBs for the transcript abundance analysis. Phylogenetic trees with pitaya candidate sequences and previously characterized sequences [30, 31] were constructed with FastTree v2 [32] (WAG + CAT model) based on alignments constructed via MAFFT v7 [33] and cleaned with pxclsq [34] to achieve a minimal occupancy of 0.1 for all alignment columns.

### Transcript abundance quantification

Quantification of transcript abundance was performed with kallisto v0.44.0 [35] using the RNAseq reads and our *Hylocereus undatus* transcriptome assembly [18]. Customized Python scripts were applied to summarize individual count tables and to compare expression values [36].

## Supplementary Information

**Additional file 1.** Phylogenetic trees of anthocyanin biosynthesis sequences and putative MYB sequences detected in our pitaya transcriptome assemblies.

**Additional file 2.** Peptide sequences of enzymes associated with the flavonoid biosynthesis of pitaya.

**Additional file 3.** Peptide sequences of putative pitaya MYBs.

## Data Availability

The datasets generated and/or analysed during the current study are available in the Bieldefeld University repository: 10.4119/unibi/2946374.
